# High Gas Sensitivity to Nitrogen Dioxide of Nanocomposite ZnO-SnO_2_ Films Activated by a Surface Electric Field

**DOI:** 10.3390/nano12122025

**Published:** 2022-06-12

**Authors:** Victor V. Petrov, Alexandra P. Ivanishcheva, Maria G. Volkova, Viktoriya Yu. Storozhenko, Irina A. Gulyaeva, Ilya V. Pankov, Vadim A. Volochaev, Soslan A. Khubezhov, Ekaterina M. Bayan

**Affiliations:** 1 Institute of Nanotechnologies, Electronics, and Equipment Engineering, Southern Federal University, 347928 Taganrog, Russia; tenirka@mail.ru; 2 Department of Chemistry, Southern Federal University, 344090 Rostov-on-Don, Russia; mvol@sfedu.ru (M.G.V.); viktoriastorojenko@gmail.com (V.Y.S.); ekbayan@sfedu.ru (E.M.B.); 3 Institute of Physical and Organic Chemistry, Southern Federal University, Stachki Av. 194/2, 344090 Rostov-on-Don, Russia; ipankov@sfedu.ru (I.V.P.); vvolochaev@sfedu.ru (V.A.V.); 4 Research Laboratory of Functional Nanomaterials Technology, Southern Federal University, Shevchenko St. 2, 344006 Taganrog, Russia; soslan.khubezhov@metalab.ifmo.ru; 5 Department of Nanophotonics and Metamaterials, ITMO University, 197101 St. Petersburg, Russia; 6 Department of Physics, North-Ossetian State University, Vatutina Str. 46, 362025 Vladikavkaz, Russia

**Keywords:** ZnO, SnO_2_, nanocomposite thin film, solid-phase pyrolysis, NO_2_ gas sensor, surface electric field

## Abstract

Gas sensors based on the multi-sensor platform MSP 632, with thin nanocomposite films based on tin dioxide with a low content of zinc oxide (0.5–5 mol.%), were synthesized using a solid-phase low-temperature pyrolysis technique. The resulting gas-sensitive ZnO-SnO_2_ films were comprehensively studied by atomic force microscopy, Kelvin probe force microscopy, X-ray diffraction, scanning electron microscopy, transmission electron microscopy, scanning transmission electron microscopy, energy dispersive X-ray spectrometry, and X-ray photoelectron spectroscopy. The obtained films are up to 200 nm thick and consist of ZnO-SnO_2_ nanocomposites, with ZnO and SnO_2_ crystallite sizes of 4–30 nm. Measurements of ZnO-SnO_2_ films containing 0.5 mol.% ZnO showed the existence of large values of surface potential, up to 1800 mV, leading to the formation of a strong surface electric field with a strength of up to 2 × 10^7^ V/cm. The presence of a strong surface electric field leads to the best gas-sensitive properties: the sensor’s responsivity is between two and nine times higher than that of sensors based on ZnO-SnO_2_ films of other compositions. A study of characteristics sensitive to NO_2_ (0.1–50 ppm) showed that gas sensors based on the ZnO-SnO_2_ film demonstrated a high sensitivity to NO_2_ with a concentration of 0.1 ppm at an operating temperature of 200 °C.

## 1. Introduction

Gas sensors based on thin-film semiconductor oxide materials are widely used due to their high sensitivity to toxic gases [1,2,3]. Metal oxides, such as tin dioxide [4], zinc oxide [5], titanium dioxide [6], cobalt oxide [7], iron oxide [8], indium oxide and others [9] are used as gas-sensitive sensor materials for monitoring the concentration of pollutants (NO_x_, CO [10]) or process gases (H_2_S, H_2_, C_3_H_8_, C_2_H_5_OH, CH_3_COCH_3_, etc. [11]). Currently of great scientific interest are the gas-sensitive properties of metal oxide nanostructure (nanotubes, nanorods, nanofilms, nanowires, etc.) materials [12,13,14].

However, despite the good gas-sensitive properties of these materials, nanostructure synthesis technology is quite difficult to apply widely in the production of gas sensors. On the other hand, thin films of nanocomposite materials are used to determine low concentrations of gases (fractions and ppm units). Excellent results are shown by thin films based on tin dioxide, with the addition of other oxides in small concentrations [9,15,16,17,18] or heterostructures [19,20]. In this case, the nature, the oxidation degree, the crystallites size, and the modifying agents’ concentration are the controlling factors of the density of surface defects and the surface area of oxide nanomaterials [21], as well as the concentration of surface oxygen [15,22]. In such materials, ZnO-ZnO, SnO_2_-SnO_2_ homostructures, and ZnO-SnO_2_ heterojunctions, as well as Schottky barriers, are formed [23]. The resulting heterojunctions lead to an improvement in the gas-sensitive properties of nanocomposite materials [24]. 

Nanocomposite materials based on SnO_2_ are obtained by various methods [22], such as magnetron sputtering methods [25], chemical technologies [26,27,28], atomic layer deposition [29], the microwave hydrothermal method [30,31], magnetron sputtering [32], spray [33,34,35] or solid-phase [36,37] pyrolysis, etc. During the preparation of this article, the authors analyzed the available publications devoted to the study of the gas-sensitive and physicochemical properties of composite materials based on SnO_2_ with a small concentration of ZnO. A literature review shows that changes in the physicochemical and gas-sensitive properties of the obtained materials are significantly influenced by the concentration of the introduced additives. 

The most important measuring results for nanocomposite ZnO-SnO_2_ films are shown in Table 1. It was found that better sensitive properties are recorded when the particle size of the gas-sensitive material is below 20–30 nm [23,29,38,39,40,41,42]. If crystallite size is higher, the sensitivity is usually lower [43,44,45]. On the other hand, the best gas-sensitive properties are shown for materials with the one component concentration equal to 10% or lower [15,29,46]. The first case can be explained by the existence of a depletion–enrichment zone comparable to the size of nanoparticles. In the second case, the situation is not fully explained. The authors of these publications, as a rule, explain a sensitivity increase by citing the presence of ZnO-SnO_2_ heterojunctions, the nanocrystalline structure, and the synergistic effect.

Additionally, there is an insufficient number of studies concerned with the influence of small concentrations of some metal oxides. The mechanism of gas sensitivity in such cases should be investigated comprehensively using X-ray photoelectron spectroscopy (XPS) [47] and high-resolution transmission electron microscopy (TEM, HRTEM).

Thus, the aim of the present work was to study the effect of small concentrations of zinc oxide (0.5–5 mol.%) on the physicochemical, electrophysical, and gas-sensitive properties of ZnO-SnO_2_ thin films. A new method of solid-phase pyrolysis was used to obtain gas-sensitive materials based on ZnO-SnO_2_ thin films. The characteristics of a gas sensor with ZnO-SnO_2_ films deposited on the MSP 632 multi-sensor platform (Heraeus Sensor Technology, Hanau, Germany) were also investigated.

## 2. Materials and Methods

### 2.1. Chemicals for Synthesis of ZnO-SnO_2_ Thin Films

Zinc acetate dihydrate (Zn(CH_3_COO)_2_·2H_2_O), stannic chloride pentahydrate (SnCl_4_·5H_2_O), an abietic acid (C_19_H_29_COOH), and 1,4-dioxane as a solvent were used as precursors for the synthesis of SnO_2_-ZnO thin films. The reagents were purchased from “ECROS”, Saint Petersburg, Russia. All chemicals used were of analytical grade or of the highest purity available. 

### 2.2. Synthesis of ZnO-SnO_2_ Thin Films

**Figure 1:**
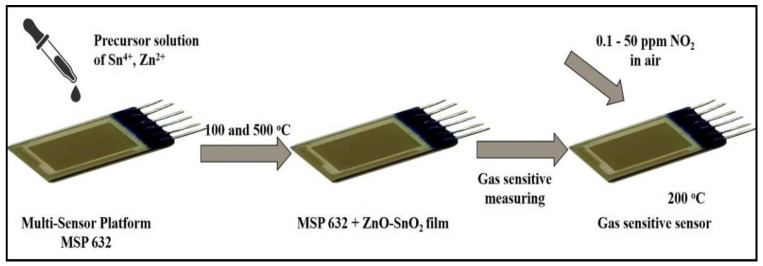
Gas sensor formation scheme.

The synthesis was carried out in two stages. In the first stage, synthesis was carried out in a melt of abietic acid. The necessary amounts of zinc and tin salts were added to the melt, after which the melt was cooled and crushed. In the second stage, the necessary amounts of organic salts were dissolved in 1,4-dioxane so that the molar ratios of tin and zinc were Zn:Sn = 0:100 (sample 0ZnO), 0.5:99.5 (sample 0.5ZnO), 1:99 (sample 1ZnO), and 5:95 (sample 5ZnO) mol.%, respectively. The resulting solution was applied three times onto polycor substrates and a multi-sensor platform. The precursor solution was deposited on the multi-sensor platform using a mechanical pipette. Each layer was dried in air and in a drying cabinet at a temperature of 100 °C. Heat treatment was carried out at 500 °C for two hours (Figure 1). The synthesis technique is described in more detail in previous studies [15,36].

To make gas sensors based on ZnO-SnO_2_ thin films, precursor was applied over a Pt counter-pin structure multi-sensor platform, MSP 632, and dried at 100 °C. Annealing at a temperature of 500 °C for two hours in air- and temperature-controlled conditions was carried out directly, using a heater and a temperature sensor MSP 632.

### 2.3. Characterization

The resulting gas-sensitive ZnO-SnO_2_ films were comprehensively studied by atomic force microscopy (AFM), Kelvin probe force microscopy (KPFM), X-ray diffraction (XRD), scanning electron microscopy (SEM), transmission electron microscopy (TEM), scanning transmission electron microscopy (STEM), energy dispersive X-ray spectrometry (EDS), and XPS. 

Electrophysical (volt-ampere characteristics; temperature dependences of electrical conductivity) and gas sensitivity measurements were estimated using an automated installation for determining the parameters of gas sensors [16]. In addition, the activation energy of conductivity (E_a_) was estimated using the Arrhenius equation (Equation (1)) [48]:

G = G_0_ exp^(−E_a_/k∙T)^ (1)

where k is the Boltzmann constant, and G_0_ the coefficient taking into account the bulk material conductivity.

Next, the temperature-stimulated conduction measurements [16,49,50] method was used to evaluate the “effective” value of the energy barrier between the grains of a nanocrystalline material.

The gas response of sensors was measured with 5–50 ppm nitrogen dioxide (NO_2_) balanced with synthetic air at operating temperatures of 200–250 °C. In the experiments, cylinders with test synthetic air and a mixture of synthetic air and NO_2_ (Monitoring LLC, Saint Petersburg, Russia) were used. The gases were injected at a flow rate of 0.3 dm^3^/min using a gas mixture generator (Microgaz F, Moscow, Russia) [16]. The response of sensor elements based on ZnO-SnO_2_ films was calculated using the following formula (Equation (2)):

S = R_g_/R_0_ (2)

where R_0_ and R_g_ are the sensors resistance in synthetic air and in a mixture of synthetic air and NO_2,_ respectively. Subsequently, the gas sensor, which showed the best gas-sensitive properties, was tested for exposure to NO_2_ with a concentration of 0.1 to 1 ppm.

## 3. Results

**Figure 2:**
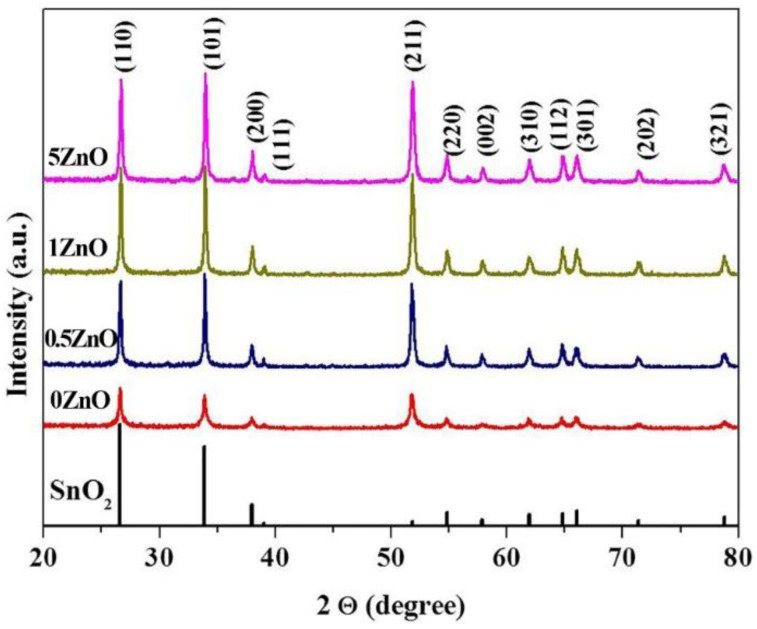
XRD patterns of synthesized film materials 0ZnO, 0.5ZnO, 1ZnO, 5ZnO.

The XRD spectra of pure and ZnO-SnO_2_ thin films are shown in Figure 2. All of the diffraction peaks are well indexed to the tetragonal structure of SnO_2_ typical for cassiterite (JCPDS: 41–1445), regardless of the amount of modifying agents. Moreover, no additional diffraction peaks of ZnO were observed, which may be due both to the small amount of it in the system and to the small size of the crystallites. No other impurity phases are observed in the XRD pattern. The XRD patterns for pure and ZnO-SnO_2_ materials show that the films have a polycrystalline microstructure.

The degree of material crystallinity was determined by XRD according to the method described in the paper [49,50]. It was found that an increase in the content of the introduced additives leads to an increase in the degree of materials’ crystallinity (57.2, 66.0, 67.0 and 67.4% for 0ZnO, 0.5ZnO, 1ZnO, and 5ZnO materials, respectively).

**Figure 3:**
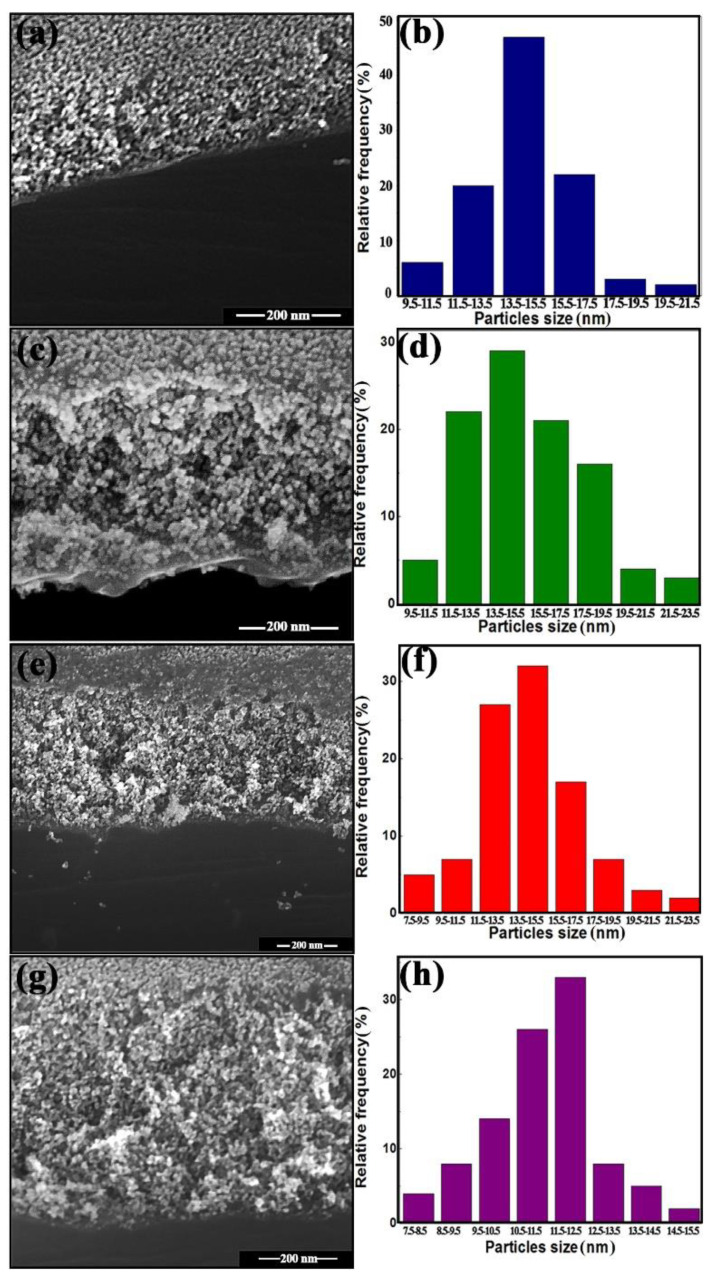
SEM microphotographs and particle size distribution of materials 0ZnO (**a**,**b**), 0.5ZnO (**c**,**d**), 1ZnO (**e**,**f**), 5ZnO (**g**,**h**).

Cross-sectional and top-view SEM images of 0ZnO (a), 0.5ZnO (c), 1ZnO (e), and 5ZnO (g) thin films are presented in Figure 3. It was found that the films are cracked, homogenous, and uniform, with a continuous distribution of grains. The type of coating obtained is an area with adjacent grains, and the thickness of the three-layer films is 160–240 nm; statistical processing of the results of the SEM analysis is shown in the histograms below. However, in SEM images, as a rule, it is difficult to determine the dimensions of agglomeration of observed nanocrystallites. Therefore, the sizes of nanocrystallites measured may, in this case, be partly overestimated.

**Figure 4:**
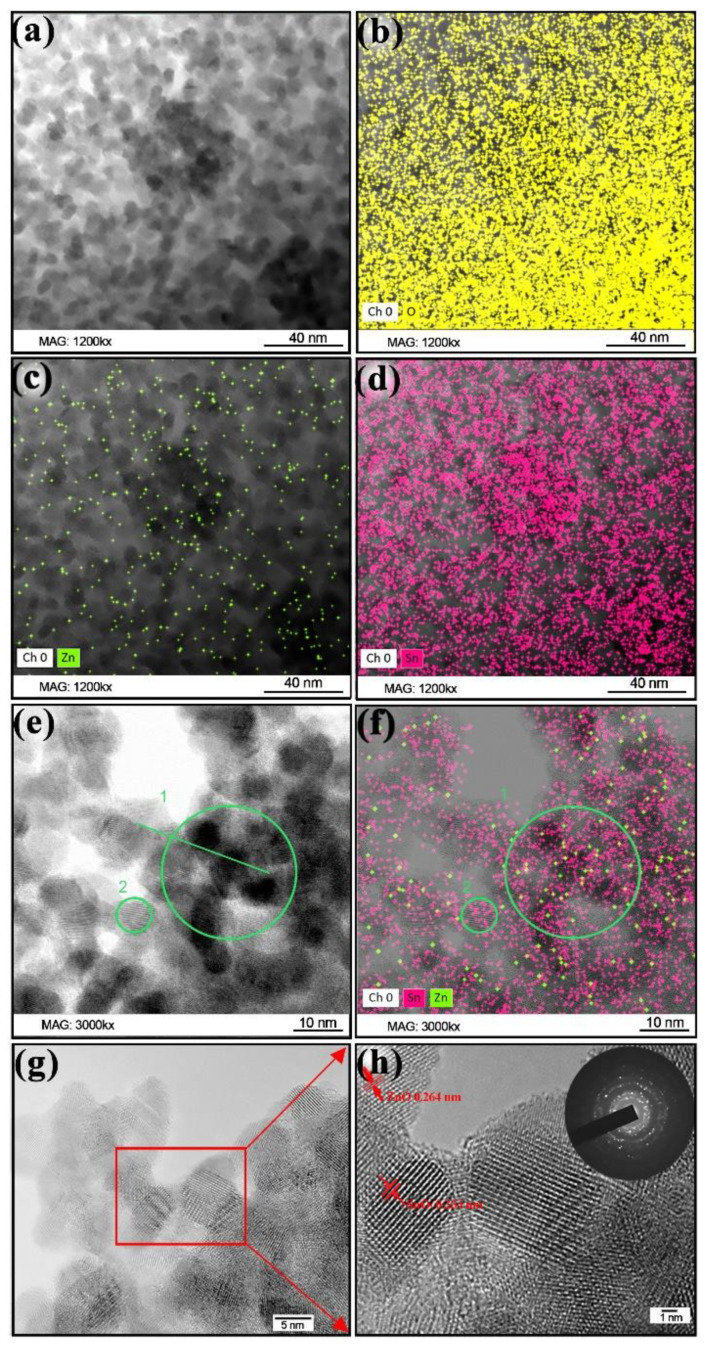
TEM characterization results of ZnO-SnO_2_ materials: (**a**,**e**,**g**) TEM images with different magnifications; (**b**–**d**,**f**) EDS elemental mapping images; (**h**) HRTEM image and insert to the right at the top—SAED images.

The 0.5ZnO thin film was investigated using the TEM method. The synthesized film material consists of many nanocrystallites with particles ranging in size from 4.5–11.5 nm (Figure 4a,e–h). Where the film is thin, SnO_2_ nanocrystallites are visible in large quantities; between these, separate ZnO nanocrystallites are located. Studies conducted using the EDX method showed that the proportion of zinc in the material, by weight, does not exceed 1% of the mass (Figure 4a–f). The mapping of the elements Zn, O, and Sn in one film’s area is shown in Figure 4b–d. It can be seen that the elements Sn, Zn, and O are evenly distributed throughout the material, without local agglomeration. The content of the elements Sn, Zn, and O is consistent with the relative amount of the elements Sn, Zn, and O in the initial solution. The TEM images show that ZnO crystallites make contact with SnO_2_ crystallites. To prove this, high resolution photos were taken; areas 1 and 2 are highlighted in Figure 4e,f. Area 2 comprises SnO_2_ nanocrystallites, which is confirmed by the EDX analysis. Area 1 consists of nanocrystallites of SnO_2_ and ZnO, which are uniformly distributed thoughout the area. Moreover, since there are significantly more SnO_2_ nanocrystallites, ZnO nanocrystallites make contact with SnO_2_ nanocrystallites.

To observe the planes of the crystal structure, images with high magnification were obtained for some sections of the film. One particle with a lattice fringe of 0.264 nm corresponds to the plane (002) of the ZnO hexagonal structure: it is shown in Figure 4h. Additionally, on this site there are areas in which it is possible to distinguish a lattice fringe of 0.334 nm, which corresponds to the plane (110) of the SnO_2_ rutile structure. Thus, it can be concluded that nanoparticles consist of ZnO and SnO_2_ crystals. Selected area electron diffraction (SAED) circles correspond to the (110), (101), (200), (211), and (112) crystal planes of SnO_2_.

**Figure 5:**
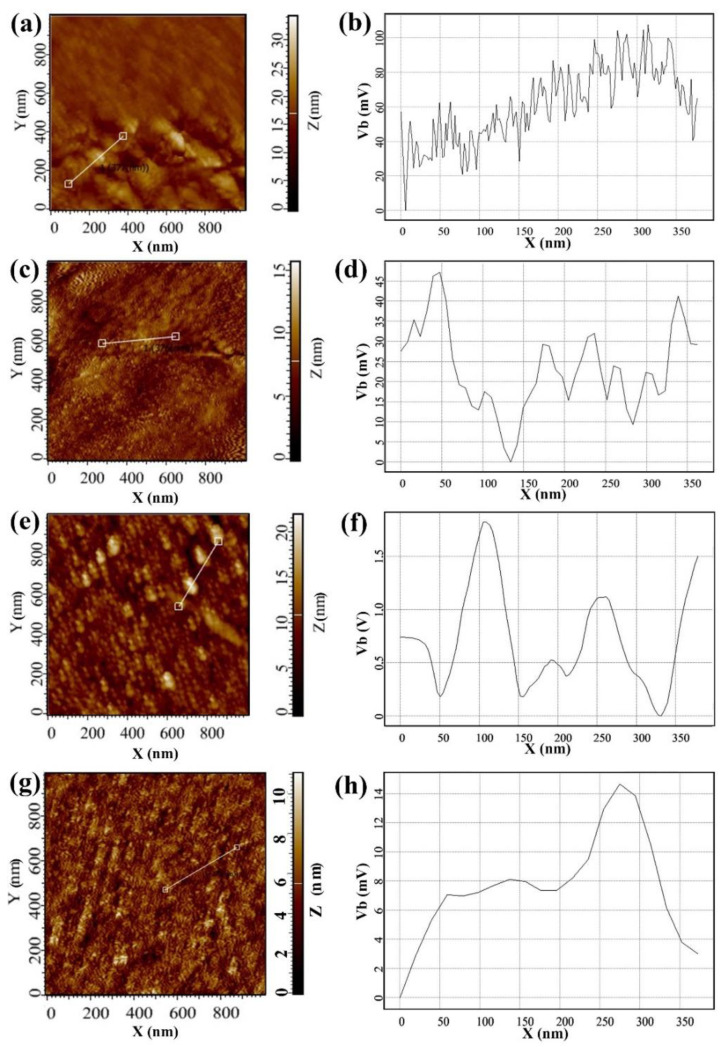
AFM images (**a**,**c**,**e**,**g**) and the corresponding distribution of the surface potential (**b**,**d**,**f**,**h**) on the films’ surface: 5ZnO (**a**,**b**), 1ZnO (**c**,**d**), 0.5ZnO (**e**,**f**), 0ZnO (**g**,**h**).

AFM and KPFM images of 0ZnO, 0.5ZnO, 1ZnO, and 5ZnO films are presented in Figure 5.

**Figure 6:**
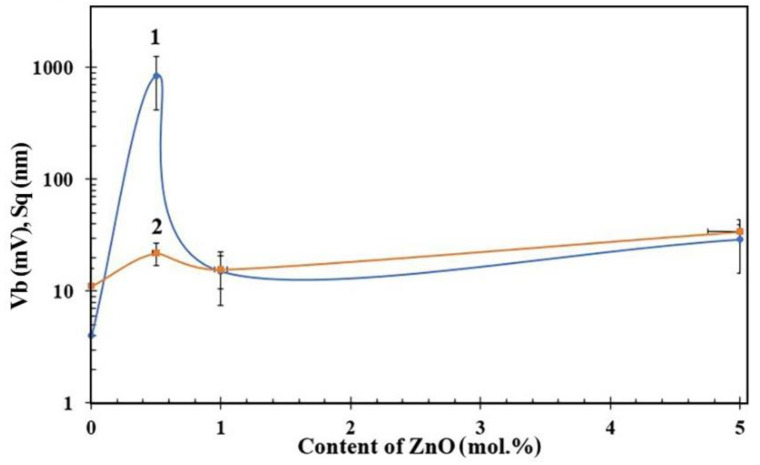
The dependence of the average values of the surface potential V_b_ (curve 1) and the roughness parameters S_q_ (curve 2) on the content of ZnO in the film.

AFM studies showed that the films have a granular structure, with a height difference of Sq of 10–35 nm (Figure 5 and Figure 6). In general, with a decrease in the content of ZnO in the film, the roughness decreases from 34 to 11 nm. However, the 0.5ZnO film surface has a higher roughness than the surface of the 0ZnO and 1ZnO films—see Figure 6 (curve 2).

KPFM studies have shown that a lowest average value of the surface potential (Figure 6 (curve 1)) of about 4 mV is characteristic of SnO_2_ film (Figure 5h). 1ZnO and 5ZnO films have similar average values of surface potential, equal to 15–30 mV (Figure 5b,d). 

However, for 0.5ZnO film, a peak value of the surface potential is observed, the average value of which reaches 845 mV, and at some points on the surface the maximum value is 1824 mV; the difference between the maximum and minimum values of V_b_ could be 1642 mV (Figure 5f and Figure S2). 

**Figure 7:**
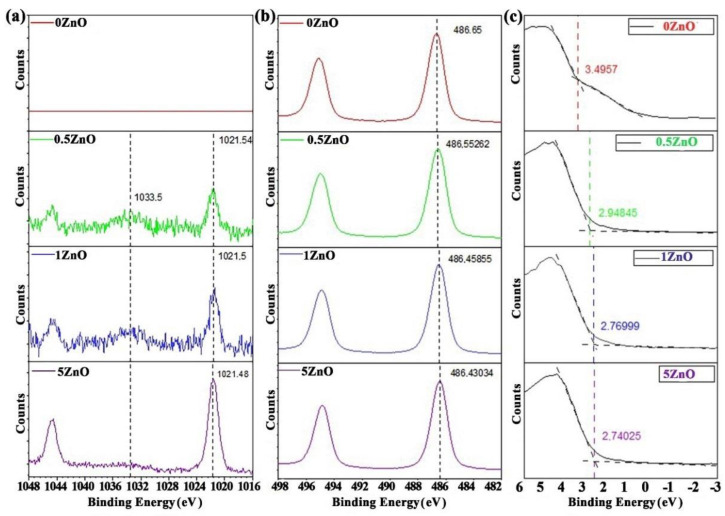
High-resolution XPS spectra of Zn2p (**a**), Sn3d (**b**), and the maximum valence band (**c**) in ZnO-SnO_2_ films.

The samples’ surface composition (Table 2) was also calculated from high-resolution XPS spectra using relative sensitivity factors from the Scofield Library. The high-resolution spectra of Zn2p, Sn3d and the valence band are presented in Figure 7. It can be seen from Figure 7b that the photoelectronic line Sn 3d for the sample 0ZnO (red curve) corresponds to SnO_2_.

**Figure 8:**
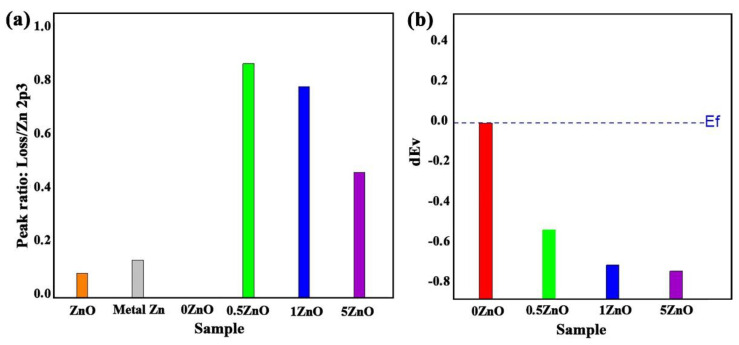
The ratio of peak intensities of zinc photoelectronic lines Zn2p3 plasmon losses (**a**), and the distribution of changes in the valence band level at the interface (**b**), in ZnO-SnO_2_ films.

Minor changes in the maximum positions of the Sn 3d photoelectronic lines for samples containing ZnO suggest the formation of a special interface at the nanocomposite oxides’ surface, which leads to a decrease in the work function. Analysis of the XPS spectra valence band edge makes it possible to evaluate and determine in more detail both the change in the maximum of the valence band (VBM), and the band gap and the Fermi level position at the phase boundary [51,52,53,54]. The formation of this interface in the ZnO-SnO_2_ system can be confirmed by analysing the high-resolution Zn2p spectra (Figure 7a). At zinc concentrations of 0.5% and 1%, the shapes of the Zn2p photoelectronic lines are very different from the case of bulk ZnO (Figure S1). In the region of binding energies of 1033.5 eV, plasmon losses, which are inherent in metallic zinc, are visible; however, the position and shape of the Zn2p3 peak exclude the presence of metallic zinc. The ratio of peak intensities of Zn2p3 photoelectronic lines’ plasmon losses for the obtained samples, as well as a comparison for pure metallic and ZnO crystalline, is shown in Figure 8a. Thus, the presence of a gap between the Fermi level and the edge of the valence band on the one hand, and the features of Zn2p photoelectronic lines on the other, completely exclude the presence of metallic zinc in the synthesized nanocomposites. The peculiarity of the Zn2p photoelectronic lines here can be linked to the formation of an interface enriched with charge carriers at the phase boundary, which leads to inelastic scattering of photoelectrons on them, and the appearance of an intense peak of plasmon losses in the region of 1033.5 eV.

The values of the valence band edge from high-resolution XPS spectra close to the Fermi level are determined using a linear approximation of the background line and the useful signal. The intersection of the obtained lines determines the value of the valence band edge, shown in Figure 7c.

From the analysis of the valence band edge, the values of the change in the valence band maximum for each sample are obtained according to the well-known formula (Equation (3)) [55]:

(3) $$\Delta E=\left(E_{\mathrm{Sn}3d}^{{\mathrm{SnO}}_{2}}-E_{\mathrm{VBM}}^{{\mathrm{SnO}}_{2}}\right)-\left(E_{\mathrm{Zn}2p}^{\mathrm{ZnO}}-E_{\mathrm{VBM}}^{\mathrm{ZnO}}\right)+\Delta E_{\mathrm{CL}}$$

where $\Delta E_{\mathrm{CL}}=E_{\mathrm{Zn}2p}^{\mathrm{ZnO}}-E_{\mathrm{Sn}3d}^{{\mathrm{SnO}}_{2}}$;

$$E_{\mathrm{Sn}3d}^{{\mathrm{SnO}}_{2}}-\mathrm{binding}\;\mathrm{energy}\;\mathrm{of}\;3d5{\;\mathrm{electrons}\;\mathrm{in}\;\mathrm{SnO}}_{2};$$

$$E_{\mathrm{VBM}}^{{\mathrm{SnO}}_{2}}-{\mathrm{valence}\;\mathrm{band}\;\mathrm{edge}\;\mathrm{value}\;\mathrm{in}\;\mathrm{SnO}}_{2}$$

$$E_{\mathrm{Zn}2p}^{\mathrm{ZnO}}-\mathrm{binding}\;\mathrm{energy}\;\mathrm{of}\;2p3\;\mathrm{electrons}\;\mathrm{in}\;\mathrm{ZnO}$$

$$E_{\mathrm{VBM}}^{\mathrm{ZnO}}-\mathrm{valence}\;\mathrm{band}\;\mathrm{edge}\;\mathrm{value}\;\mathrm{in}\;в\;\mathrm{ZnO}$$

Valence band edge values and the binding energy of core levels for ZnO2p3/2 and SnO_2_ 3d5/2 are presented in Table 3.

The distribution of the change in the valence band level at the interface for each sample is presented below in Figure 8b.

**Figure 9:**
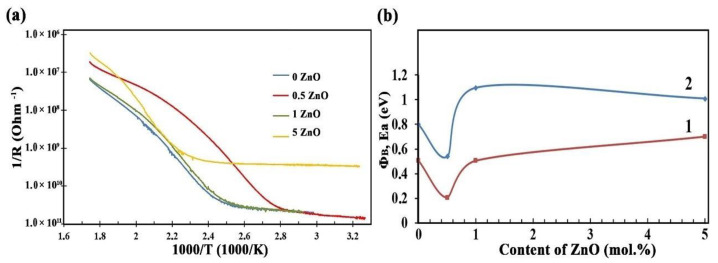
(**a**) Temperature dependence of gas sensors’ reverse resistance and (**b**) dependences of the activation energy of the conductivity E_a_ (curve 1) and the potential barrier (curve 2) of ZnO-SnO_2_ films on the content of ZnO.

The dependence of reverse resistance on reverse temperature for all samples has a characteristic form for metal oxide semiconductors (Figure 9).

The activation energy of conductivity (E_a_), calculated by the Arrhenius equation (Figure 9b, curve 1), shows a sharp (by 2–2.5 times) decrease in the E_a_ and the potential barrier Φ_B_ for 0.5ZnO films. The E_a_ decreases from 0.51 eV to 0.2 eV, and the Φ_B_ from 1.1 eV to 0.54 eV. 

**Figure 10:**
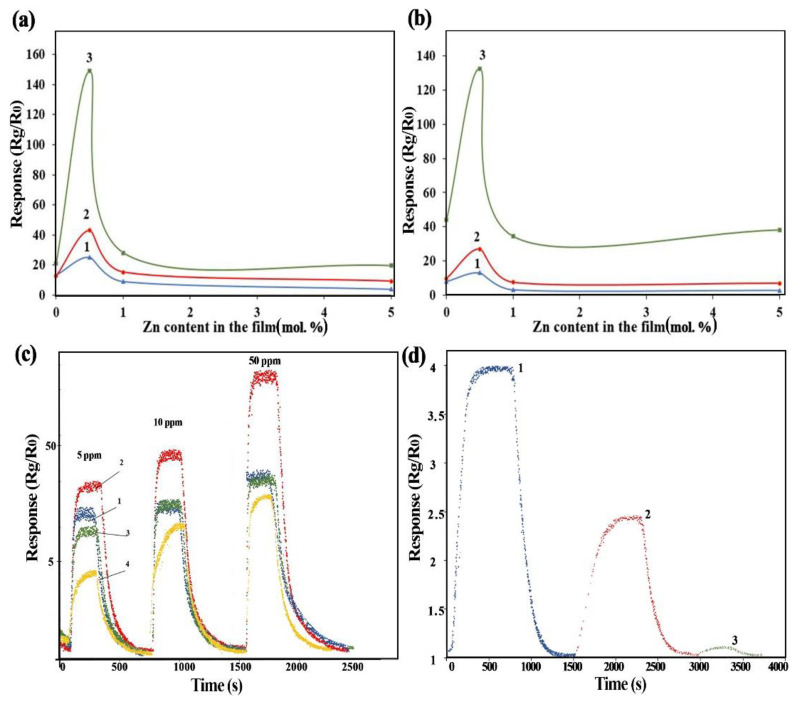
The dependences of the ZnO-SnO_2_ gas sensors response on exposure to NO_2_ concentrations of 5 ppm (1), 10 ppm (2) and 50 ppm (3) at 200 °C (**a**), 250 °C (**b**) and (**c**) the normalized response of gas sensors based on ZnO-SnO_2_ films (**c**): 0ZnO (1), 0.5ZnO (2), 1ZnO (3) and 5ZnO (4) when exposed to NO_2_ concentrations of 5, 10, and 50 ppm, and (**d**) 0.5: 99.5 when exposed to NO_2_ concentrations of 0.1, 0.5, and 1.0 ppm at an operating temperature of 200 °C.

The results of the gas sensitivity study of ZnO-SnO_2_ films at 200 °C and 250 °C are shown in Figure 10. The typical response of gas sensors when exposed to NO_2_ with concentrations of 5, 10, and 50 ppm is shown in Figure 10c.

It is shown (Figure 10c) that, when exposed to NO_2_ concentrations of 5, 10, and 50 ppm, a gas sensor based on a 0.5ZnO-99.5SnO_2_ film is approximately 1.5–9 times more sensitive to NO_2_ compared to gas sensors based on ZnO-SnO_2_ films with other compositions. For exposure to NO_2_ with a concentration of 50 ppm and an exposure temperature of 200 °C, the sensor’s response S is equal to 146. The evaluation of the response time t_resp_ shows (Table 4) that for this sensor, when exposed to 5 ppm NO_2_, t_resp_ is 144 s and 80 s at operating temperatures of 200 and 250 °C, respectively.

A gas sensor based on a 0.5ZnO-99.5SnO_2_ film has a higher sensitivity; it was therefore investigated for exposure to NO_2_ with concentrations from 0.1 to 1 ppm. The sensor response of this gas sensor is shown in Figure 10d. The response value, when exposed to NO_2_ with a concentration of 1.0 ppm, is 3.9, and, when exposed to NO_2_ with a concentration of 0.1 ppm, is 1.1.

Thus, gas sensors based on a ZnO-SnO_2_ film, synthesized using a solid-phase pyrolysis technique with a ratio of 0.5ZnO, have a range of measured concentrations of NO_2_ equal to 0.1–50 ppm, with a response time of 80–144 s, and an operating temperature of 200 °C.

To test the selectivity of the sensor based on a 0.5ZnO-99.5SnO_2_ film to other gases, studies were conducted with acetone, ethanol, ammonia, and water molecules, with a concentration of 200 ppm at an operating temperature of 200 °C. The sensor response was 1.1 for acetone, 1.25 for ethanol, 1.76 for ammonia, and 1.1 for water. It can be seen that, in comparison with the response value for nitrogen dioxide with a concentration of 50 ppm (146), these values do not exceed 1.2%.

## 4. Discussion

It is known that the valence band edge for pure SnO_2_ is 3.5 eV [54,55]. This is also confirmed by our measurements for the material ZnO:SnO_2_ = 0:100 (Figure 8, Table 3). For 0.5ZnO film materials or for other ZnO-SnO_2_ materials, the energy level of the valence band edge decreases to 2.95 eV. Further decrease in the valence band edge value is slower—up to 2.77 and 2.74 eV for 1ZnO and 5ZnO materials, respectively. Thus, the energy of the valence band edge in ZnO-SnO_2_ films decreases with an increase in the ZnO concentration.

Potential barriers are formed at the interface of SnO_2_-SnO_2_ homostructures and the ZnO-SnO_2_ heterojunction. Measured by temperature-stimulated conduction measurements, the value of the potential barrier Φ_B_ for the SnO_2_ film was 0.80 eV (Figure 9b). This potential barrier is formed due to the adsorption of oxygen molecules on the surface of SnO_2_ nanocrystals, and their subsequent ionization with the formation of O_2_^−^ ions [33,56,57,58,59,60].

**Figure 11:**
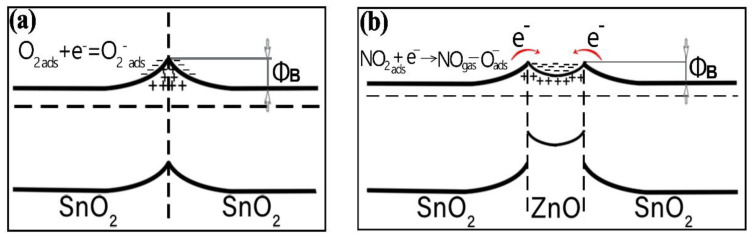
Flat band scheme of crystallite contacts for SnO_2_-SnO_2_ (**a**) and SnO_2_-ZnO-SnO_2_ (**b**).

The contact of two SnO_2_ nanocrystallites is shown in Figure 11a. It is well known that, because of this contact, a depletion region is formed in SnO_2_ nanocrystallites [59,60,61].

The study of the gas sensor based on the film material 0.5ZnO-99.5SnO_2_ showed that the value of Φ_B_ sharply decreases to 0.47 eV (Figure 9b). With a further increase in the ZnO concentration (1ZnO and 5ZnO), Φ_B_ becomes significantly higher—1.1 and 1.0 eV, respectively. The mechanism of a sharp decrease in Φ_B_ in the gas sensor based on the 0.5ZnO-99.5SnO_2_ film is shown in Figure 11b. It is known that the work function of ZnO (5.2–5.3 eV) [62,63] is higher than that of SnO_2_ (4.8–4.9 eV) [61,64]. In addition, the Fermi level of SnO_2_ is closer to the vacuum level, in relation to the Fermi level of ZnO. When ZnO and SnO_2_ crystallites are in contact with each other, electrons will transfer from tin dioxide to zinc oxide (Figure 11). The value of the potential barrier at the SnO_2_-ZnO-SnO_2_ heterojunction will decrease in this case (Figure 9b). According to TEM studies (Figure 4c), individual ZnO crystallites come into contact with SnO_2_ crystallites, so ZnO becomes highly enriched with charge carriers. This fact is also confirmed by XPS studies: plasmon losses are observed on Zn2p3 zinc photoelectronic lines (Figure 7a). The same effect leads to the appearance of regions with large local surface potential Vb. This is confirmed by our KPFM measurements (Figure 5c and Figure 6b). On the surface of the 0.5ZnO film, the average value of the surface potential is 845 mV, and in some places reaches values of 1824 mV (Figure 5c). For film materials with a higher content of zinc oxides (1ZnO and 5ZnO), the concentration of ZnO crystallites becomes higher, which leads to a decrease in the average value of the surface potential to 15–30 mV. The existence of high values of the V_b_ in 0.5ZnO-99.5SnO_2_ films leads to the formation of a strong surface electric field. The electric field contributes to the reduction of the Φ_B_ between nanocrystallites. In this regard, E_a_ also decreases in the 0.5ZnO-99.5SnO_2_ film. In addition, it can be noted that the surface potential affects the morphology of the film’s surface (Figure 6). 

As can be seen from Figure 10, gas sensors based on the film material 0.5ZnO-99.5SnO_2_ show a peak response to NO_2_ exposure, compared to other samples.

The reason for this sharp increase in response may, on the one hand, be a decrease in the potential barrier Φ_B_ [65]. On the other hand, however, the reason for the sharp increase in the response of gas sensors based on film material 0.5ZnO-99.5SnO_2_ is, in our opinion, the high surface potential formed due to the contact of ZnO and SnO_2_ crystallites. The analysis of TEM images (Figure 4) shows that the distance between the crystallites is no more than 0.1 nm. At such values, distances, and potentials, there is a surface electric field with a strength of 2 × 10^7^ V/cm. It is known that an electric field with a strength of up to 3 × 10^7^ V/cm can exist in oxide materials if their thickness is up to 4 nm [66,67]. The presence of a surface electric field is supported by the response decreasing from 200 °C to 250 °C (Figure 10a,b), since it is well known that an increase in temperature leads to a decrease in the influence of the electric field. The effect of the electric field influence on the atoms’ diffusion over the surface, and in the volume of solids, has been well studied [68,69]. 

As shown in [70], the surface electric field of this magnitude, created by charged adsorption centers, significantly affects the mechanism of the interaction of polar gas molecules with the gas-sensitive material’s surface.

It is known that the NO_2_ molecule has a dipole moment 0.29–0.33 D [71,72,73]. In this case, the interaction energy of the polar adsorbate molecule with the charged adsorption center (Q_aa_) can be estimated using the following formula (Equation (4)) [74]:

(4) $$Q_{\mathrm{aa}}=-N_{a}\times \frac{e\times V\times \mu }{z_{0}^{2}}$$

where e is the electron charge; V is the valence of the adsorbent ion; μ is the dipole moment of the adsorbate molecule; z_0_ is the equilibrium distance between the adsorbate molecule and the charged adsorption centre; and Na is the Avogadro constant.

Calculations show that if z_0_ is equal to 0.2 nm, the Q_aa_ for the NO_2_ molecule is 31–35 kJ/mol. 

At the same time, we have shown that a sufficiently strong surface electric field (Es), of magnitude (1–2) × 10^7^ V/cm, is created at the crystallite boundary of tin and zinc oxides. The processes of adsorption and dissociation of NO_2_ molecules are activated by a strong surface electric field. NO_2_ molecules receive an additional field-induced dipole moment (µ_i_), the magnitude of which depends on the polarizability of the α molecule. It is known that the NO_2_ molecule has a high polarizability (α), equal to 1.8 × 10^−24^ cm^3^ [67]. 

When the amount of polarizability increases, the surface electric field effect on the molecule also increases. The energy of the adsorbate–adsorbent interaction (Q_aa_) increases by the value of the induction component (Q_i_) associated with the occurrence of the induced dipole moment. The value of µ_i_ can be estimated using the formula (Equation (5)) [75].

μ_i_ = α × E_s_. (5)

Calculation of the total dipole moment (μ′ = μ + µ_i_) shows that μ′ can be equal to 0.47–0.51 D.

In addition, the adsorbate molecule will be affected by orientation polarization, in accordance with the direction of the electric field, which depends on the magnitude of the molecule’s dipole moments [75]. The energy of the orientation influence (Q_or_) of the field is equal to (Equation (6)):

Q_or_ = 0.5 × μ′ × E_s_, (6)

The total energy of the adsorbate adsorbent (Q_aa_′) interaction between the adsorption centre and the NO_2_ molecule is calculated by the formula (Equation (7)):

Q_aa_′ = (Q_aa_ + Q_i_ + Q_or_). (7)

The calculation results show that, for an NO_2_ molecule with high polarizability, the Q_aa_′ can increase to 57–60 kJ/mol, that is, almost twofold. Such energy may already be enough to initiate the process of dissociation of gas molecules, since the activation energy of the molecule’s dissociation is half of the molecule dissociation energy [76,77]. The latter circumstance means that dissociation of the NO_2_ molecule is possible. These arguments do not contradict previously published works [78,79]. Thus, adsorption of NO_2_ molecules, with their subsequent dissociation, can occur (Equation (8)):

(8) $$NO_{2,gas}+e\to NO_{2,ads}^{-}\to O_{}^{-}{}_{ads}+NO_{gas},$$

A strong surface electric field is a powerful activating factor, as is ultraviolet radiation. In the presence of these factors, forced ionization of adsorbed molecules can occur (Equation (9)) [80]:

(9) $$NO_{{}_{2}gas}+O_{2}^{-}+2e\to NO_{2}^{-}+2O^{-}$$

As can be seen, these reactions can lead to a stronger response to NO_2_ molecules.

A comparative analysis of the gas sensitivity to NO_2_ of nanocomposite ZnO-SnO_2_ structures presented in Table 1 and in this paper shows that the size of nanostructures (nanocrystallites) is essential for high gas sensitivity to low concentrations of NO_2_ in the air. In all works, the nanostructures of the gas-sensitive material are formed by different technologies, and have sizes from 5 to 17 nm [15,29,30,40,41,42]. In addition, when the operating temperature of the gas sensor decreases, the response and recovery times increases [30,81]. The optimal value of the operating temperatures for sensors with low concentrations of nitrogen dioxide is, in our opinion, in the range of 150–250 °C, in order to achieve the optimal value of response times of 80–140 s. When analyzing these articles, the gas-sensitive properties of sensors based on ZnO-SnO_2_ films are similar. 

The characteristics of NO_2_ sensors based on other materials are presented in Table 5.

Sensors operating at room temperature have long response/recovery times [82,83,84,89,90,91,92,93]. The advantage of such sensors is that there is no need for heating to operating temperature, and they can be used indoors. Since gas sensors are used for NO_2_ monitoring outside, in a wide temperature range, sensors operating at 200 °C are recommended for analysis unification. Sensors with response/recovery times in the range of 30–180 s have a more complex technology for forming a gas-sensitive layer [86,87,88,93]. Some gas-sensitive materials are unstable over time, in particular metal sulfides [83,85,86].

Thus, it can be seen that many materials used in gas sensors of the resistive type have similar gas-sensitive characteristics. The choice of gas-sensitive materials is determined by the following requirement: “simple technology—high-quality, reproducible and fast response to a given concentration.” The solid-phase pyrolysis method proposed in this work and in our other works [15,36] satisfies this requirement. Using the described method, it is possible to obtain composite nanoscale materials of various compositions with high values of gas-sensitive characteristics. In this case, by selecting the composition of mixed ZnO-SnO_2_ oxides, this technology allows for the formation of heterostructures with a strong surface electric field, thereby “fine-tuning” the gas-sensitive properties.

## 5. Conclusions

Nitrogen dioxide gas sensors were formed on the MSP 632 multi-sensor platform. The gas-sensitive element of the sensor is a ZnO-SnO_2_ film with thickness 60–200 nm. The film materials were formed using a new low-temperature pyrolysis technique; the zinc oxide content was from 0.5 to 5 mol.%. 

Comprehensive studies of the materials’ physicochemical, structural, electrophysical, and other properties were carried out using modern research methods. It was shown that all ZnO-SnO_2_ films are composite with crystallite sizes of 4–30 nm and have a cassiterite structure. Zinc oxide nanocrystallites are evenly distributed in the structure of ZnO-SnO_2_ films. 

This leads to the creation, in the 0.5ZnO-99.5SnO_2_ film, of a surface potential of up to 1800 mV. This is because of the existence of a strong surface electric field with a strength of (1–2) × 10^7^ V/cm.

When NO_2_ molecules approach the surface of the ZnO-SnO_2_ film, a strong surface electric field promotes the induction of a dipole moment in the molecule. As a result, this leads to an increase in the energy of the adsorbate–adsorbent interaction between the adsorption center and the NO_2_ molecule, and an activation of the molecules’ dissociation process. The response of the gas sensor based on 0.5ZnO film is 1.5–9 times higher compared to sensors based on other ZnO-SnO_2_ films.

Thus, it was shown that the gas sensor based on film material 0.5ZnO-99.5SnO_2_ is very promising for the detection of NO_2_ gas at concentrations of 0.1–50 ppm; it has a response time equal to 80–144 s and an operating temperature of 200 °C. 

## Figures and Tables

**Table 1:** Parameters and characteristics of gas-sensitive materials.

№	Material (Composition, Structure)	Technology	Particle Size, nm	The Formula for Response Calculation (Gas Concentration)	Operating Temperature, °C	Response Value	Response/Recovery Time, s	Reference
H_2_								
1	SnO_2_-ZnO (0.9:0.1)	Electrospinning method	15	R_a_/R_g_, (0.1–10 ppm)	300	168.6	10^3^/10^3^	[23]
2	Sn-ZnO	Spray pyrolysis	8–14	∆R/R, (500 ppm)	400	200	50/80	[38]
3	ZnO-SnO_2_	Chemical synthesis	50–90	(R_a_ − R_g_) × 100% R_a_ (10,000 ppm)	150	90%	60/80	[39]
NO_2_								
4	SnO_2_-ZnO	Pulse laser depostion	10–20	R_g_/R_a_ (3.2 ppm)	180	100	240/480	[40]
5	SnO_2_-ZnO (5:95)	Chemical technologies	5–10	R_g_/R_a_ (0.5–1.0 ppm)	150	48	100/101	[41]
6	7% Sb-SnO_2_/ZnO	Microwave hydrothermal	10	R_g_ − R_a_ R_a_ (1000 ppb)	300	9.5	16/-	[29]
7	ZnO-SnO_2_ (1:1)	Wet chemical method	11–17	R_g_ − R_a_ R_a_ (500 ppb)	20	13.4	420/480	[42]
8	ZnO-SnO_2_	Magnetron sputtering	10	R_a_/R_g_ (5 ppm)	100	26.4	20/45	[30]
9	SnO_2_-ZnO (1: 99)	Solid phase pyrolysis	13–14	R_g_/R_a_ (5 ppm)	200	4.5	300/400	[15]
Ethanol								
10	SnO_2_-ZnO (1:1)	Combined deposition,	20–40	R_a_/R_g_ (200 ppm)	300	4.69	72/-	[43]
11	Au-doped SnO_2_-ZnO (1:0.5; 1:1; 0.5:1)	Electrospin coating	5–10	R_a_/R_g_ (100 ppm)	300	90	130/	[44]
12	SnO_2_:ZnO= (3:1; 1:1; 1:3)	Chemical deposition,	2800	(R_a_ − R_g_) × 100% R_a_ (24 ppm)	275	53%	150/-	[45]
13	SnO_2_/ZnO core/shell	The thermal evaporation SnO_2_ NWs and the spray-coating of ZnO	150	R_a_/R_g_ 100 ppm	450	15.9	215/-	[46]

**Table 2:** The surface composition of ZnO-SnO_2_ films.

Materials	C 1s	O 1s	Sn3d5	Zn2p3
0ZnO	34.15	44.85	21.00	0
0.5ZnO	27.36	45.64	26.53	0.47
1ZnO	33.35	40.73	24.47	1.45
5ZnO	33.39	41.05	22.55	3.01

**Table 3:** Valence band edge values and binding energy of core levels.

Materials	VBM, (eV)	Zn2p3, (eV)	Sn3d5, (eV)	ΔE, (eV)	$\Delta E_{CL},\;(\mathrm{eV})$
0ZnO	3.49	-	486.65	0	-
0.5ZnO	2.95	1021.54	486.55	−0.55	534.99
1ZnO	2.77	1021.50	486.45	−0.73	535.05
5ZnO	2.74	1021.48	486.43	−0.76	535.05

**Table 4:** Response time of ZnO-SnO_2_ films to NO_2_ exposure.

Materials of Gas Sensor	t_resp_. (s), When Exposed to NO_2_ Gas by Working Temperature					
	200 °C			250 °C		
	5 ppm	10 ppm	50 ppm	5 ppm	10 ppm	50 ppm
0ZnO	67	60	58	87	70	86
0.5ZnO	144	144	240	80	86	244
1ZnO	151	126	108	85	90	162
5ZnO	394	388	87	126	170	552

**Table 5:** Gas-sensitive characteristics of NO_2_ sensors based on other materials.

Material	Method	Gas Concentration, ppm	Operating Temperature, °C	Sensitivity	Response/Recovery Time	Reference
Single wall carbon nanotubes—Mn-porphyrin	Chemical technologies and Langmuir–Blodgett technique	2.5	100	38%	9 min/--	[82]
WS_2_	Drawing on paper	0.8	Room temperature	42%	5.2 min/19 min	[83]
Zn_(0.5)_Fe_(0.5)2_O_4_	Method of sol–gel auto combustion	5	90	0.54%	100 s/100 s	[84]
MoS_2_/ZnO	Wet chemical method	5	Room temperature	3050%	211 s/1000 s	[85]
Thioglycolate- capped CdS quantum dots	Electrochemical method	0.011	Room temperature	17%	<30 s/<30 s	[86]
WO_3_ nanofiber	Electrospinning	3	90	100	125 s/231 s	[87]
SnO–Sn_3_O_4_	Solvothermal process	0.5	75	63.4	87 s/178 s	[88]
Au/pr-In_2_O_3_	Ultrasonic-Spray Pyrolysis	5	100	~300	30 min/doesn’t recover	[89]
Al-doped NiO	RF-sputtered	0.2	200	2.7	20 min/40 min	[90]
voltage activation rGO	Chemically reducing GO water dispersion	0.05	Room temperature	5	2.1 min/28 min	[91]
porous polythiophene (PTh) films	Plasma jets polymerization technique	0.25	Room temperature	21%	1250 s/2500 s	[92]
polypyrrole/Fe_2_O_3_	One-step hydrothermal technique	0.1	50	128%	150 s/879 s	[93]
0.5ZnO-99.5SnO_2_	Solid-phase low-temperature pyrolysis	0.5	200	2.5	80 s/90 s	This work

## Data Availability

Not applicable.

## Supplementary Materials

The following supporting information can be downloaded at https://www.mdpi.com/article/10.3390/nano12122025/s1, Figure S1: The XPS spectra of pure zinc and its oxide, Figure S2: Distribution of the surface potential on the film surface 0.5ZnO.
