# Supplementary material for: High Gas Sensitivity to Nitrogen Dioxide of Nanocomposite ZnO-SnO2 Films Activated by a Surface Electric Field
Source: Nanomaterials (Basel). 2022 Jun 12;12(12):2025. doi: 10.3390/nano12122025 (PMC9230884; doi:10.3390/nano12122025)
Supplement: Supplementary file 1 [file nanomaterials-12-02025-s001.zip › nanomaterials-1746065-supplementary.pdf]

nanomaterials

Supplementary Material 1

High gas sensitivity to nitrogen dioxide of nanocomposite $ZnO-SnO_2$ films activated by a surface electric field

Victor V. Petrov*[1], Aleksandra P. Ivanishcheva [1], Maria G. Volkova[2], Viktoriya Yu. Storozhenko[2], Irina A. Gulyaeva[1], Ilya V. Pankov[3], Vadim A. Volochaev[3], Soslan A. Khubezhov[4,5,6], Ekaterina M. Bayan[2]

High Gas Sensitivity to Nitrogen Dioxide of Nanocomposite $ZnO-SnO_2$ Films Activated by a Surface Electric Field

Victor V. Petrov [1,*], Alexandra P. Ivanishcheva [1,*], Maria G. Volkova [2], Viktoriya Yu. Storozhenko [2], Irina A. Gulyaeva [1], Ilya V. Pankov [3], Vadim A. Volochaev [3], Soslan A. Khubezhov [4,5,6] and Ekaterina M. Bayan [2]

[1] Institute of Nanotechnologies, Electronics, and Equipment Engineering, Southern Federal University, 347928 Taganrog, Russia; a.starnikova@mail.ru (A.P.I.); tenirka@mail.ru (I.A.G.)
[2] Department of Chemistry, Southern Federal University, 344090 Rostov-on-Don, Russia; mvol@sfedu.ru (M.G.V.); viktoriastorojenko@gmail.com (V.Y.S.); ekbayan@sfedu.ru (E.M.B.)
[3] Institute of Physical and Organic Chemistry, Southern Federal University, Stachki Av. 194/2, 344090 Rostov-on-Don, Russia; ipankov@sfedu.ru (I.V.P.); vvolochaev@sfedu.ru (V.A.V.)
[4] Research Laboratory of Functional Nanomaterials Technology, Southern Federal University, 347922 Shevchenko St. 2, 344006 Taganrog, Russia; soslan.khubezhov@metalab.ifmo.ru
[5] Department of Nanophotonics and Metamaterials, ITMO University, 197101 St. Petersburg, Russia
[6] Department of Physics, North-Ossetian State University, Vatutina Str. 46, 362025 Vladikavkaz, Russia
* Correspondence: vvpetrov@sfedu.ru (V.V.P.); a.starnikova@mail.ru (A.P.I.) ; Tel.: +7-863-437-1624 (V.V.P.)

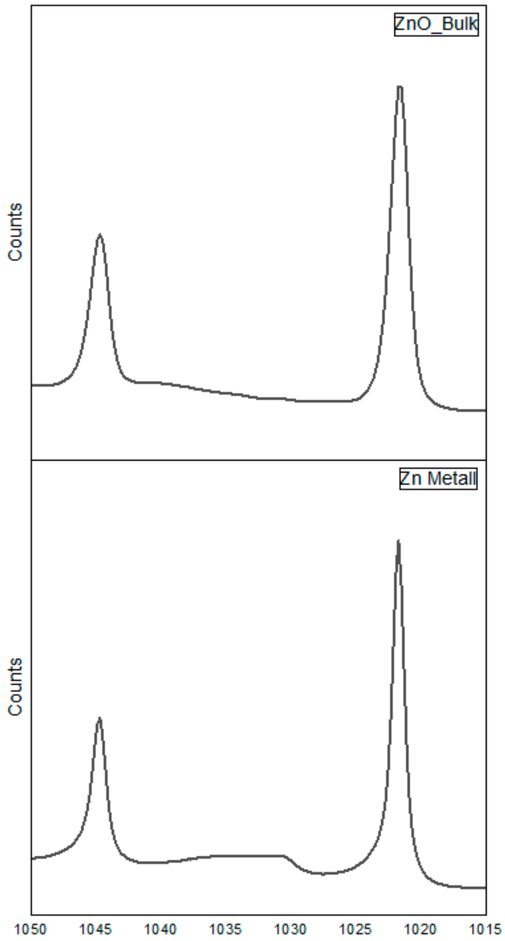

Figure S1. The XPS spectra of pure zinc and its oxide.

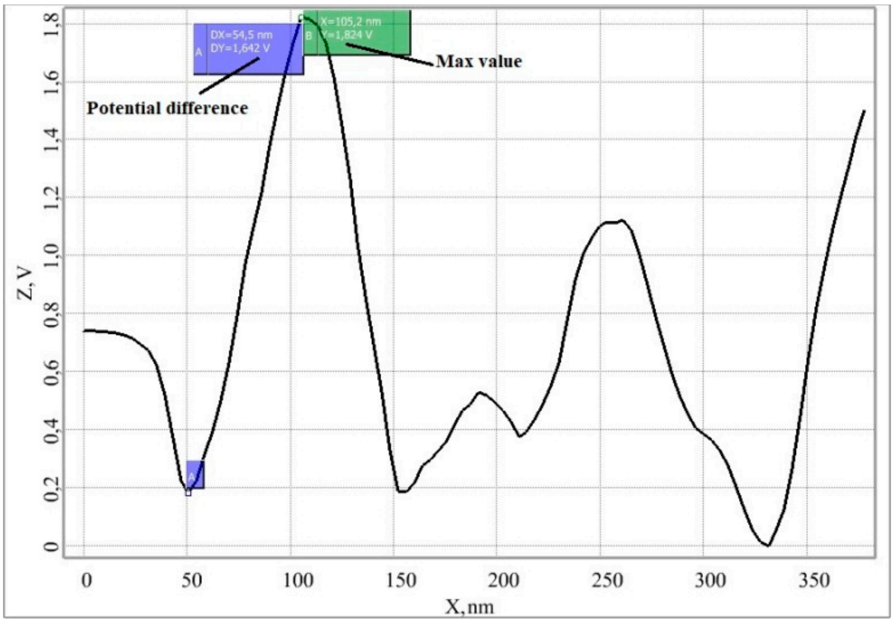

Figure S2. Distribution of the surface potential on the film surface 0.5ZnO.
